# Design and Analysis of a Continuously Tunable Low Noise Amplifier for Software Defined Radio

**DOI:** 10.3390/s19061273

**Published:** 2019-03-13

**Authors:** Aayush Aneja, Xue Jun Li

**Affiliations:** Department of Electrical and Electronic Engineering, Auckland University of Technology, Auckland 1010, New Zealand; aayush.aneja@aut.ac.nz

**Keywords:** green radio, software defined radio, low noise amplifier, transformer network, tuning transistor, phase shifter

## Abstract

This paper presents the design and analysis of a continuously tunable low noise amplifier (LNA) with an operating frequency from 2.2 GHz to 2.8 GHz. Continuous tuning is achieved through a radio frequency impedance transformer network in the input matching stage. The proposed circuit consists of four stages, namely transformer stage, tuning stage, phase shifter and gain stage. Frequency tuning is controlled by varying output current through bias voltage of tuning stage. The circuit includes an active phase shifter in the feedback path of amplifier to shift the phase of the amplified signal. Phase shift is required to further achieve tunability through transformer. The LNA achieves a maximum simulated gain of 18 dB. The LNA attains a perfect impedance match across the tuning range with stable operation. In addition, it achieves a minimum noise figure of 1.4 dB.

## 1. Introduction

The number of wireless standards has increased rapidly in the last decade. Together with advancements in integrated circuit (IC) technologies, it has proliferated research in multiband radio systems. As such, Software Defined Radio (SDR) has gained popularity due to its ability to handle multiple bands through a single system. In addition, SDR can enable green radio networks (GRNs) by adapting the operating frequency to a band with less interference. Next-generation SDR systems possess the capability to eliminate external processing hardware and implement associated software instead for digital signal processing and digitization [1]. The literature on this topic focuses on improving the flexibility of SDR with specific attention on the front end.

In general, the design requirements for an SDR low noise amplifier (LNA) are yet to be standardized. An LNA is the first active circuit in the receiver front-end chain of an SDR. It should primarily have a high voltage gain, low noise figure (NF) and wideband impedance matching. In case of a band-limited SDR, the LNA should support reconfigurable multiband operation [2]. Additionally, a stable and linear operation is desired at all frequencies of operation. It is challenging for a reconfigurable LNA to achieve a desired narrowband bandpass response at individual center frequencies [3] so that out-of-band interferers in the operational bandwidth can be filtered out. Designing a reconfigurable input matching network with narrowband response is highly efficient as compared to a wideband matching network, where noise and interference from adjacent bands are difficult to suppress [4].

Reconfigurable LNAs for SDRs are broadly divided as switchable LNAs and tunable LNAs. For an SDR with limited bandwidth, tunable LNAs with continuous or discrete tuning are preferred. Tunable LNAs are further divided as input tuning LNAs and output tuning LNAs.

**Figure 1:**
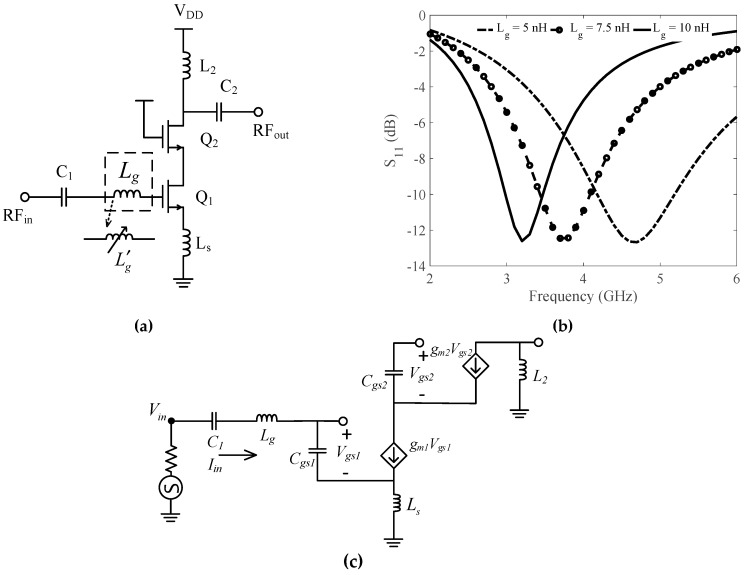
(**a**) Conceptual representation of a conventional input tuning LNA with *L_g_* (**b**) Corresponding varying S_11_ for different values of *L_g_*. (**c**) Small signal equivalent of conventional LNA.

Input tuning is referred to the capability to reconfigure the frequency response of the input impedance by variation of one or more elements in the input matching stage [5]. On the contrary, output tuning refers to variation of elements in the output matching or loading stage to vary the response of output impedance. Output tuning LNAs [6,7] usually implement a wideband input matching network and a tunable output load. Wideband matching in such LNAs requires careful frequency planning to filter out interference. Input tuning LNAs implement a wideband load and a tunable input matching. It is important to select an appropriate topology for designing an input tuning LNA. Common gate (CG) topology was implemented in [8,9] to get the wideband response and stable operation. However, the topology is not desired for designing an input tuning LNA due to the dependence of gain and NF on transconductance *g_m_*. Common Source (CS) LNA with inductive degeneration increases the real part of input impedance. It further improves the overall gain and noise matching of the circuit. Figure 1 illustrates the concept of input tuning LNA using a conventional source degenerated narrowband LNA. Replacing gate inductor *L_g_* with a variable inductor $L_{g}^{\prime }$ provides reconfigurable input impedance matching and therefore variable minimum input return loss (S_11_) at different center frequencies. Nevertheless, implementing an LNA with $L_{g}^{\prime }$ will consume a large on chip area and hysteresis due to tunable inductor would degrade the overall Figure-of-Merit (FOM) of the LNA.

For input tuning LNAs, impedance transformer [10,11], tunable floating inductor [5] and switched inductors [12] were explored. The design in [10] proposed a continuously tunable LNA (CTLNA) to accommodate a large bandwidth with relatively less on-chip area. To achieve this, an ideal amplifier was applied to the feedback of *L_g_*. Consequently, *L_g_* can be scaled by a factor that is proportional to the gain of the amplifier. However, adding additional amplifier increases the noise in the circuit and power consumption as well.

For output tuning LNAs [13], variable capacitor [6,14], switched capacitors [15,16] and varactor [17] were implemented as output load. Switched capacitors and inductors provide agile discrete tuning, but lead to substantial increase in chip area and manufacturing costs. In addition, tunable active inductors were explored to overcome the low *Q* of inductors and their large area consumption on chip. However, drawbacks associated with active inductors are higher power consumption, high noise and nonlinearity. As compared to input tuning LNAs, output tuning LNAs are more susceptible to process variations. They also need additional passives for designing a wideband input matching network. Furthermore, less input tuning LNAs were reported in the literature as compared to output tuning LNAs [18].

FPGA based reconfigurable amplifiers [19] have been explored for lower frequency applications other than SDR. Transformer based matching networks [11,20] feature an interesting alternative with a wider tuning range [21] and reduced power consumption. In this paper, we provide a comprehensive design and analysis of an input tuning LNA that implements a physical radio frequency (RF) transformer to dynamically tune the input impedance. The LNA achieves a tunable input matching and a wideband output matching from 2.2 GHz to 2.8 GHz. The organization of the paper is as follows—Section 2 presents the motivation. Section 3 discusses the analysis and design of the proposed CTLNA. Section 4 presents the overall architecture of CTLNA with analysis of input impedance, gain and NF. Section 5 discusses simulation results and finally Section 6 concludes the paper.

## 2. Motivation

A conventional narrowband LNA as shown in Figure 1a consists of a gate inductor and a source inductor in its input matching stage. The input impedance of this LNA can be derived from the small signal equivalent circuit shown in Figure 1c. Applying KVL to the circuit, the total input voltage *V_in_* is

(1) $$V_{in}=j\omega L_{g}I_{in}+\frac{I_{in}}{j\omega C_{1}}+\frac{I_{in}}{j\omega C_{gs1}}+\left(I_{in}+g_{m}V_{gs1}\right)j\omega L_{s}$$

(2) $$\frac{V_{in}}{I_{in}}=j\omega \left(L_{g}+L_{s}\right)+\frac{1}{j\omega C_{1}}+\frac{1}{j\omega C_{gs1}}+\frac{j\omega L_{s}g_{m}V_{gs1}}{I_{in}}$$

(3) $$V_{gs1}=\frac{I_{in}}{j\omega C_{gs1}}$$

From Equations (2) and (3), input impedance $Z_{in}$ of conventional narrowband LNA can be given as

(4) $$Z_{in}=\left(j\omega \left(L_{g}+L_{s}\right)-j\left(\frac{1}{\omega C_{gs1}}+\frac{1}{\omega C_{1}}\right)\right)+\frac{g_{m1}L_{s}}{C_{gs1}}$$

where $g_{m1}$ and $C_{gs1}$ are the transconductance and gate-source capacitance of transistor Q_1_, respectively. The resonant frequency of input matching network depends on $C_{gs1}$, $L_{g}$ and source inductor $L_{s}$. The resonant frequency at which $Z_{in}$ is real can be determined as

(5) $$f_{0}=\frac{1}{2\pi \sqrt{\left(L_{s}+L_{g}\right)\cdot C_{x}}}$$

where *C*_x_ is equivalent capacitance of $C_{gs1}$ and *C*_1_. It can be concluded from Equations (4) and (5) that *Z_in_* and *f*_0_ can be made tunable by either varying *L_s_* and *L_g_*. Since, Re(*Z_in_*) is directly proportional to *L_s_*, replacing *L_g_* with $L_{g}^{\prime }$ could be a viable solution. Nevertheless, additional amplification stage is required to make *L_g_* tunable or floating, which increases the die-area, implementation costs, NF and power consumption.

A feasible and efficient solution is to replace *L_g_* with a physical RF impedance transformer, whose secondary winding can act as a variable inductor. The secondary inductance can be changed through an additional circuit connected to the primary winding of transformer network. Using switching circuits with primary winding and inductive-capacitive resonant networks would not provide continuous tuning. Moreover, additional switching circuits shall increase power consumption and NF of the circuit. In this paper, we propose a CTLNA with tunable input matching network, comprising of a physical RF impedance transformer network. Input impedance can be varied to achieve minimum S_11_ at each center frequency by changing the magnitude of current flowing through secondary winding of the transformer network. This can be achieved through magnetic coupling between primary and secondary windings of the transformer. Furthermore, the proposed LNA architecture comprises of an inductive load that provides a wideband response in the tuning range. This approach is expedient to maintain small area, continuous tuning and avoiding noise contributing elements in the signal path.

## 3. Proposed Circuit Topology

**Figure 2:**
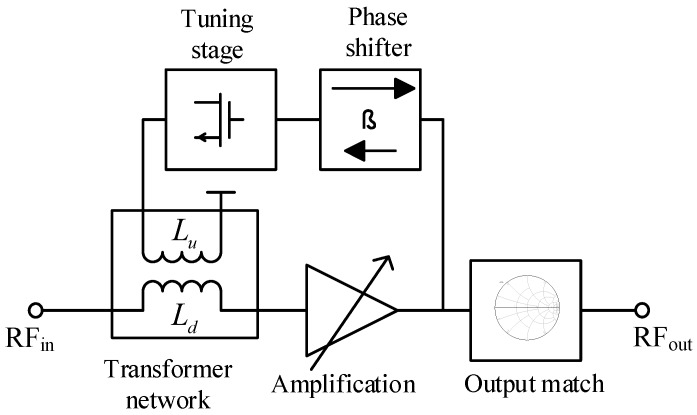
Conceptual block of proposed CTLNA.

Figure 2 shows the block representation of the proposed CTLNA which consists of four different stages. The first stage is the input matching stage that consists of an input capacitor C_1_ and a physical transformer. The second stage consists of a phase shifter network that comprises of two CG transistors connected in parallel to a CS transistor to get a relative 0° or 180° phase shift between the currents through primary and the secondary windings of the transformer. The third stage consists of tuning transistor whose bias voltage *V_tune_* can be varied to get the desired tunability. Finally, the fourth stage is the amplification stage that achieves a tunable wideband gain when *V_tune_* is varied. For better understanding of the proposed circuit topology, design and synthesis of each stage is described as follows.

### 3.1. Transformer Network

**Figure 3:**
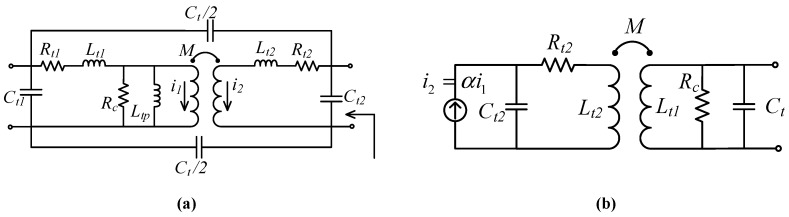
(**a**) Physical transformer equivalent circuit for designed CTLNA (**b**) simplified transformer model for calculations.

The transformer in the input stage is an RF impedance transformer. One end of its primary winding *L_u_* is connected to the output of tuning transistor, while the other end is connected to the voltage supply *V_DD2_* = 1.3 V. The secondary winding *L_d_* is connected to the input transistor via a DC bias network. If *L_d_* is considered as a variable inductor as shown in Figure 2, then scaling its value will provide a 50 Ω impedance matching at different center frequencies. The design utilizes a similar concept by implementing an RF impedance transformer in place of a variable inductor. Therefore, frequency reconfigurability can be achieved if current passing through *L_d_* can be changed. The magnetism property of transformer can be utilized [22] to change current through *L_d_*. However, the currents *i*_1_ and *i*_2_ through *L_u_* and *L_d_* must have a relative phase shift *ϕ* of either 0° or 180° to allow continuous frequency tunability. This is because the RF impedance transformer circuit, shown in Figure 3a, provides a 50 Ω impedance match at a phase difference of 0° or 180° and the impedance is purely real at *ϕ* = 0°.This can be substantiated by deriving the relationship between transformer’s input impedance $Z_{inT}(\omega )$ and ϕ. From the simplified transformer network shown in Figure 3b and $Z_{inT}(\omega )$ can be given as

(6) $$\frac{1}{Z_{inT}\left(s\right)}=\frac{1}{sL_{1}+s\alpha M}+\frac{1}{R_{c}}+sC_{t}$$

(7) $$\frac{1}{Z_{inT}(s)}=\frac{R_{c}+sL_{t1}+s\alpha M+s^{2}C_{t1}L_{t1}R_{c}+s^{2}\alpha MR_{c}C_{t1}}{R_{c}\left(sL_{t1}+\alpha M\right)}$$

Inverting Equation (7) and substituting *s* = *jω*, $Z_{inT}\left(\omega \right)$ is

(8) $$Z_{inT}(\omega )=\frac{j\omega R_{c}\left(L_{t1}+\alpha M\right)}{R_{c}\left(1-\omega^{2}C_{t}\left(L_{t1}-\alpha M\right)\right)+j\omega \left(L_{t1}+\alpha M\right)}$$

where $\alpha =i_{2}/i_{1}$ is the ratio of primary and secondary winding currents in the transformer network, *M* is the mutual inductance, $L_{t1}$ is primary leakage, $C_{t}$ is interwinding capacitance and $R_{c}$ is core loss resistance. Inductances $L_{t1}$ and $L_{t2}$ correspond to inductances $L_{u}$ and $L_{d}$ in the implemented transformer network and given as.

(9) $$L_{t1}=L_{p}\left(\frac{1}{k}-1\right)$$

(10) $$L_{t2}=\frac{L_{t1}}{N^{2}}$$

where *k* is the coefficient of coupling and *N* is the turns ratio. Due to phase difference between $i_{2}$ and $i_{1}$, $i_{2}=\beta i_{1}e^{-j\phi }$ where β is the gain and $\alpha =\beta e^{-j\phi }$ [22]. Therefore, Equation (8) can be expanded as

(11) $$Z_{inT}(\omega )=\frac{j\omega R_{c}\left(L_{t1}+\beta e^{-j\phi }M\right)}{R_{c}\left(1-\omega^{2}C_{t}\left(L_{t1}-\beta e^{-j\phi }M\right)\right)+j\omega \left(L_{t1}+\beta e^{-j\phi }M\right)}$$

**Figure 4:**
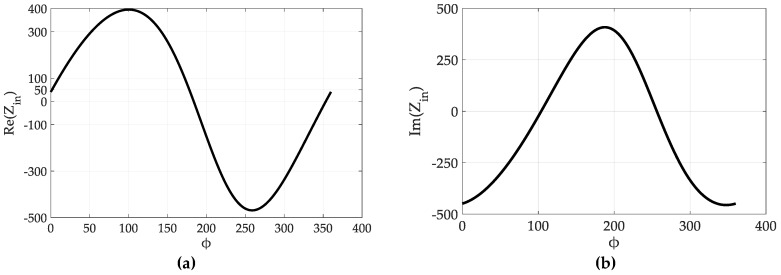
(**a**) Re(Zin), (**b**) Im(Zin) as a function of ϕ.

Substituting values for variables in Equation (11) as *R_c_* = 0.91 Ω, β = 1, *ω* = 2π*f*, *f* = 3 GHz, *L*_t1_ = 3.37 nH, *M* = 0.5 nH, *C* = 995 fF and plotting Re(Zin) vs. ϕ from 0° to 360°, we can verify that |Re(Zin)| = 50 Ω at 0° and 180° as shown in Figure 4a, despite the fact that our transformer model is different to that in [22].

Additionally, Im(Zin) is maximum at *ϕ* = 180° which leads to a phase mismatch between *i*_1_ and *i*_2_; however, the desired relative phase shift between *i*_1_ and *i*_2_ is 0° for continuous tuning. Moreover, the amplified signal is an inverted version of input signal. A possible solution is a phase shifter circuit that can provide a phase mismatch of 0° to ensure that currents $i_{1}$ and $i_{2}$ are in phase. The resonant frequency $f_{T}$ of transformer can be determined as

(12) $$f_{T}=\frac{1}{2\pi (L_{t1}\pm \alpha M)C_{t}}$$

The transformer’s coefficient of coupling *k* is related to *M* as $M=k\sqrt{L_{u}L_{d}}$. A lower value of *k* would result in lower *M* and less sensitivity of transformer network to large frequency variation and current mismatch. Therefore, the value of *k* was kept low to achieve the desirable input match. Table 1 summarizes the design parameters for the transformer network.

### 3.2. Phase Shifter

**Figure 5:**
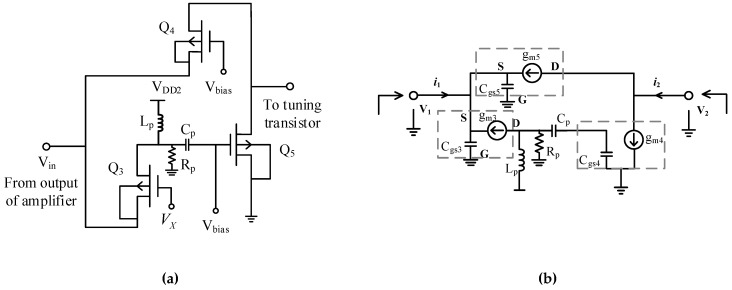
(**a**) Implemented PS circuit, (**b**) equivalent small signal model [23].

The circuit implements a conventional active phase shifter (APS) [23] to shift the phase of the amplified signal. The APS receives the amplifier output and is applied in the feedback path of the circuit. The circuit embeds two CG transistors in parallel to a CS transistor. Figure 5a shows the schematic of adapted APS circuit with a conventional topology. The designed circuit is capable of providing a phase shift of more than 90°, thereby leading to elimination of phase mismatch between complex currents $i_{1}$ and $i_{2}$. A simplified small signal equivalent circuit to illustrate the conventional APS operation is shown in Figure 5b. According to [23], $Y_{21}$ in admittance matrix for the APS is given as:

(13) $$Y_{21}(\omega )=\frac{i_{2}}{v_{1}}=-g_{m3}\cdot \left(\frac{\frac{1}{L_{p}\left(C_{p}+C_{gs4}\right)}-j\omega \frac{1}{\left(C_{p}+C_{gs4}\right)}\left(\frac{g_{m3}g_{m4}}{g_{m5}}-\frac{1}{R_{p}}\right)-\omega^{2}}{\frac{1}{L_{p}\left(C_{p}+C_{gs4}\right)}+\frac{j\omega }{R_{p}\left(C_{p}+C_{gs4}\right)}-\omega^{2}}\right)$$

Transformation of $Y_{21}\left(\omega \right)$ to $S_{21}\left(\omega \right)$ can be expressed as

(14) $$S_{21}\left(\omega \right)=\left(\frac{\frac{2g_{m5}}{g_{m3}+g_{m5}}\sqrt{Z_{inPS}\cdot Z_{outPS}}-Z_{inPS}-\frac{j\omega Z_{inPS}}{\omega_{T}}Z_{outPS}}{\frac{1}{g_{m3}+g_{m5}}+Z_{inPS}+\frac{j\omega Z_{inPS}}{\omega_{T}}Z_{outPS}}\right)\cdot \left(\frac{\frac{1}{L_{p}\left(C_{p}+C_{gs4}\right)}-\frac{j\omega }{R_{p}\left(C_{p}+C_{gs4}\right)}-\omega^{2}}{\frac{1}{L_{p}\left(C_{p}+C_{gs4}\right)}+\frac{j\omega }{R_{p}\left(C_{p}+C_{gs4}\right)}-\omega^{2}}\right)$$

**Figure 6:**
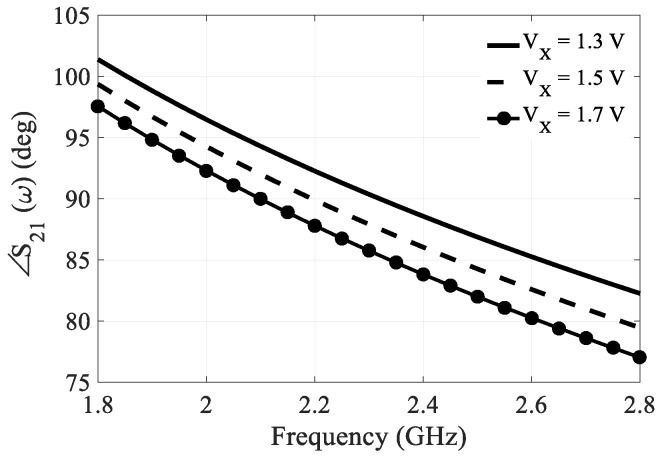
Frequency at different values of $V_{X}$.

From Equation (14), the phase of $S_{21}\left(\omega \right)$ can be derived as

(15) $$∠S_{21}\left(\omega \right)=-\tan^{-1}\left(\frac{\frac{\omega }{\omega_{T}}Z_{inPS}}{\frac{1}{g_{m3}+g_{m5}}+Z_{inPS}}\right)\;-2\tan^{-1}\left(\frac{\omega }{R_{p}\left(\frac{1}{L_{p}}-\left(C_{p}+C_{gs4}\right)\omega^{2}\right)}\right)$$

where $Z_{inPS}$ and $Z_{outPS}$ are the input impedance and the output impedance of APS, respectively. It can be concluded from Equation (15) that phase of *S*_21_(ω) depends upon inductor *L_p_* and capacitor *C_p_*. The shift in phase of the signal with constant signal amplitude is accomplished by variation in inductance or capacitance of the resonant circuit. The values of *L_p_* and *C_p_* for 2.2 to 2.8 GHz band are 17.5 nH and 10 pF, respectively. Figure 6 shows variation of phase of *S*_21_(ω) of APS with *V_X_*. The circuit provides a more than 90° phase shift in our desired frequency range.

### 3.3. Tuning Stage

**Figure 7:**
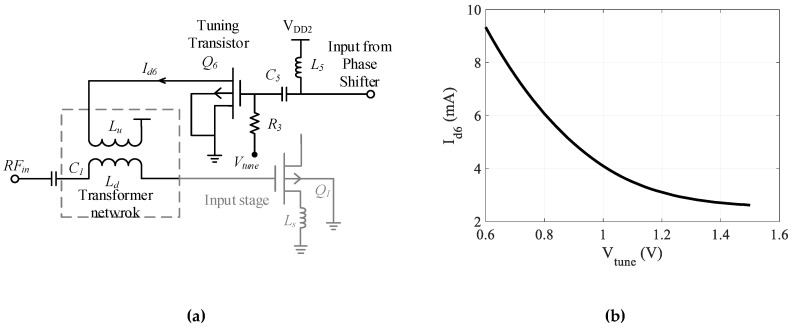
(**a**) Tuning stage of proposed CTLNA (**b**) variation of $I_{d}$ with $V_{tune}$.

Figure 7a shows the tuning stage of the designed CTLNA. It consists of a CS transistor biased with a positive gate voltage through a bias resistor. The CS transistor is placed in the feedback path and the input to its gate terminal is a phase shifted signal from the output of APS circuit. The output drain terminal is connected to one end of primary winding *L_u_* of transformer network in the input stage. Varying the bias voltage ($V_{tune}$) of tuning transistor Q_6_ continuously leads to incessant variation in its drain current $i_{d6}$. This further leads to variation in current $i_{1}$ flowing through $L_{u}$ and resultantly in *α* and *β*. Figure 7b shows the variation of $i_{d6}$ with $V_{tune}$. The resultant change in $Z_{inT}$ (depends on *β*) varies the input impedance of CTLNA, leading to continuous tunability.

## 4. Circuit Analysis

**Figure 8:**
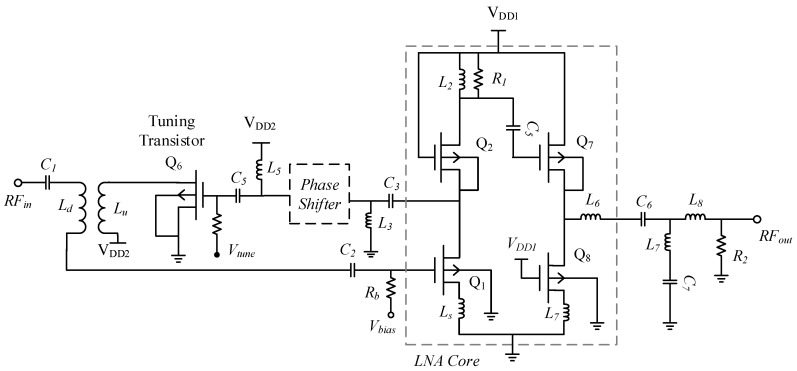
Complete CTLNA architecture.

Figure 8 shows the complete architecture of designed CTLNA with source degeneration and cascode topology. The cascode topology increases the circuit’s AC resistance and aids in augmenting the gain. Inductive degeneration increases the real part of input impedance. The primary consideration while designing a CTLNA is to determine the band of operation. *C_t_*, *L_p_* and *k* values in transformer network are then selected to focus the desired operating band that ranges from 2.2 GHz to 2.8 GHz. One end of primary winding of the transformer in input stage is terminated with output from the tuning transistor, while the other end is connected to voltage supply. Input capacitor *C*_1_ resonates with *L_d_* to achieve a continuously tunable impedance matching at different center frequencies. Continuous tuning shall only take place when $i_{1}$ and $i_{2}$ are in phase. The input of APS circuit is connected to the drain of Q_1_ via *L*_3_–*C*_3_ network. It provides a phase mismatch of 0° between the currents $i_{1}$ and $i_{2}$ through *L_u_* and *L_d_*. The output of APS is fed to gate of Q6 whose drain terminal further connects to *L_u_* to achieve tunable input matching.

A resistance $R_{b}$ is also added for the purpose of providing DC bias to the input transistor Q_1_. For simplicity, a fixed inductor $L_{2}$ was adopted in the output loading section of LNA to achieve a wideband gain. A large resistance $R_{1}$ is added in parallel to $L_{2}$ for improving LNA stability at different frequencies and DC voltage gain.

### 4.1. Input Impedance

The input stage of the proposed CTLNA consists of capacitor *C*_1_ and the transformer network. Secondary inductor *L_d_* can be considered as a tunable inductor $L_{g}^{\prime }$ that replaces *L_g_* in Figure 1 to achieve tunable input impedance. As *L_d_* cannot be directly varied, magnetic coupling can be utilised to vary the input impedance of LNA. Since $Z_{inT}\left(\omega \right)$ depends on *α*, the input impedance of CTLNA in Figure 8 is derived as

(16) $$Z_{in}(\omega )=j\left(\omega L_{s}+\omega L_{d}\pm \omega \alpha M-\frac{1}{\omega C_{gs_{1}}}-\frac{1}{\omega C_{1}}\right)+\frac{g_{m_{1}}L_{s}}{C_{gs_{1}}}$$

(17) $$f_{op}=\frac{1}{2\pi \sqrt{(L_{s}+L_{d}\pm \alpha M)C_{x}}}$$

where *C*_x_ is equivalent capacitance of $C_{gs1}$ and *C*_1_. Equation (17) shows that $f_{op}$ depends on constants *L_d_*, *L_s_*, *M*, $C_{gs1}$ and variable *α*. The value of *α* can be varied by changing $V_{tune}$ that controls $i_{d6}$ and $i_{1}$. Note that the real part of input impedance depends on *L_s_* and can be changed by varying *L_s_* only. Its value has been selected to ensure that $Z_{in}$ is matched to the source. The quality factor of input matching network $\left(Q_{in}\right)$ is one of the primary elements used to determine the bandwidth of network. For the designed CTLNA, $Q_{in}$ can be expressed as

(18) Qin=XLR=ωLRe(Zin)=ω(Ls+Ld±αM)(gm1LsCgs1)

where $X_{L}$ and *R* are imaginary and real part of input impedance, respectively. From (17) and (18), $Q_{in}$ can be simplified as

(19) $$Q_{in}=\frac{1}{\omega \left(g_{m1}L_{s}+C_{gs1}\right)}$$

It can be concluded from (19) that the bandwidth and $f_{op}$ of CTLNA increases as *Q_in_* becomes smaller.

### 4.2. Gain

**Figure 9:**
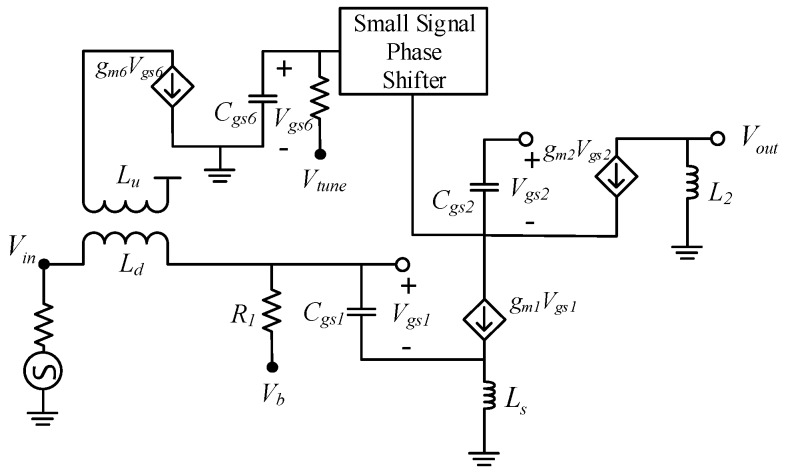
Simplified small signal model of CTLNA for gain analysis.

Gain of designed CTLNA can be derived similar to a narrowband LNA shown in Figure 1. However, in this case, the input gate inductor *L_g_* is replaced with a transformer based variable inductor *L_d_* and its impedance $Z_{inT}\left(\omega \right)$ depends on *M* and *α*. The output loading network is similar to a conventional load. The low noise voltage gain for CTLNA can be derived from its small signal model of input and amplification stage shown in Figure 9. For cascode LNAs, since all transistors are the same,

(20) $$g_{m1}=g_{m2}=2k_{n}\left(V_{gs}-V_{T}\right)$$

where $k_{n}$ is conduction parameter, $g_{m2}$ is the transconductance of the transistor Q_2_ and $V_{T}$ is the threshold voltage of implemented Philips MOS transistor. The small signal voltage gain $A_{v}$ of an LNA is defined as

(21) $$A_{v}=\frac{V_{out}}{V_{in}}$$

(22) $$V_{out}=i_{d}Z_{out}=g_{m1}V_{gs1}\cdot Z_{out}$$

(23) $$V_{in}=V_{gs1}(1+Z_{in})$$

Substituting Equation (16) in Equation (23), *V_in_* expands to

(24) $$V_{in}=V_{gs1}\left(1+j\omega \left(L_{s}+L_{d}+\alpha M\right)+\frac{1}{j\omega C_{x}}+\frac{g_{m}L_{s}}{C_{gs1}}\right)$$

Also,

(25) $$Z_{out}=\frac{g_{m2}L_{2}}{C_{gs2}}$$

From Equations (22) and (25),

(26) $$V_{out}=\frac{g_{m1}g_{m2}L_{2}}{C_{gs2}}.V_{gs1}$$

Finally, substituting Equations (24) and (26) in Equation (21), $A_{v}$ can be derived as

(27) $$A_{v}=\frac{V_{out}}{V_{in}}=\frac{j\omega g_{m1}g_{m2}L_{2}C_{x}C_{gs1}}{\left(C_{gs1}\left(1-\omega^{2}C_{x}\left(L_{d}+L_{s}+\alpha M\right)\right)+j\omega C_{x}\left(1+g_{m1}L_{s}\right)\right).\left(C_{gs2}\right)}$$

where $C_{gs2}$ is the gate-source capacitance of the transistor Q_2_. Equation (27) substantiates that $A_{v}$ for the designed CTLNA depends on *α* and eventually on $V_{tune}$. Hence the gain can also be tuned continuously in the desired band by sweeping $V_{tune}$ from 0.5 V to 1.5 V.

### 4.3. Noise Figure

**Figure 10:**
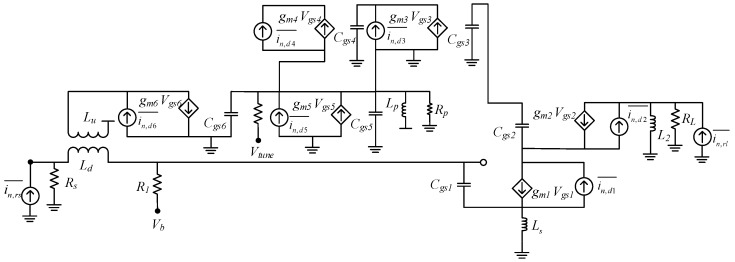
Noise equivalent model of designed CTLNA.

Figure 10 shows the noise equivalent model for the designed circuit. NF for the proposed CTLNA can be quantified by deriving its noise factor *F*. The main noise source in the circuit is thermal noise and all passives in the circuit are considered as ideal. Considering that there are multiple noise sources in the circuit, it would be rather impractical to evaluate *F* without detailed noise model for all noise sources. Therefore, an expression for output noise current due to all noise sources is calculated. The short circuit noise current due to source is

(28) $$i_{sc,R_{s}}=\frac{g_{m_{1}}V_{n,s}}{j\omega C_{gs1}R_{s}+Z_{in}^{\prime }}\cdot \omega_{z}$$

and

(29) $$Z_{in}^{\prime }=1-\omega^{2}C_{gs1}(L_{s}+L_{d})+j\omega g_{m1}L_{s}\;,\;\;\omega_{z}=\frac{g_{m2}}{g_{m\;Eq}+j\omega C_{Eq}}$$

where $g_{m\;Eq}=g_{m2}+g_{m3}+g_{m4}$ and $C_{Eq}=C_{gs2}+C_{gs3}+C_{gs4}$, $\omega_{z}$ is the zero introduced due to noise effect from other transistors Q_2_, Q_3_ and Q_4_ in parallel and $V_{n,s}$ is the noise voltage at source. The short circuit noise current due to thermal drain noise of transistor Q_1_, Q_2_ in amplification stage is

(30) $$\begin{array}{l} i_{sc,d1}=\left(i_{n,d1}-\frac{j\omega g_{m_{1}}L_{s}i_{n,d1}}{j\omega C_{gs1}R_{s}+Z_{in}^{\prime }}\right)\cdot \left(-\omega_{z}\right)\\ i_{sc,d2}=i_{n,d2}\left(1-\omega_{z}\right) \end{array}$$

where $i_{n,d1}$ and $i_{n,d2}$ are drain noise currents of Q_1_ and Q_2_. The transistors Q_3_, Q_4_ and Q_5_ in the PS circuit also contribute to the overall NF of CTLNA. Therefore, short circuit noise current due to drain noise of Q_3_, Q_4_ and Q_5_ is

(31) $$\begin{array}{l} i_{sc,d3}=i_{n,d3}\cdot \left(-\omega_{z}\right)\\ i_{sc,d4}=i_{n,d4}\cdot \left(-\omega_{z}\right)\\ i_{sc,d5}=i_{n,d5}\cdot \left(\frac{g_{m2}}{g_{m6}}\right) \end{array}$$

The short-circuit noise current due to drain noise of tuning transistor Q_6_ is

(32) $$i_{sc,d6}=\frac{j\omega g_{m1}i_{n,d6}}{j\omega C_{gs1}R_{s}+Z_{in}^{\prime }}\cdot \left(-\omega_{z}\right)$$

and due to load is

(33) $$i_{sc,rl}=i_{n,rl}$$

where $i_{n,d3}$, $i_{n,d4}$, $i_{n,d5}$, $i_{n,d6}$ are the noise currents of transistors Q_3_, Q_4_, Q_5_ and Q_6_ and $i_{n,rl}$ is the noise current of load resistance *R_L_*. Using (28) to (33), *F* for the proposed LNA can be derived as

(34) $$F=\frac{1+\sum_{x=1}^{6}\bar{i_{n,dx}^{2}}+\bar{i_{sc,rl}^{2}}}{\bar{i_{sc,rs}^{2}}}$$

## 5. Results and Discussion

**Figure 11:**
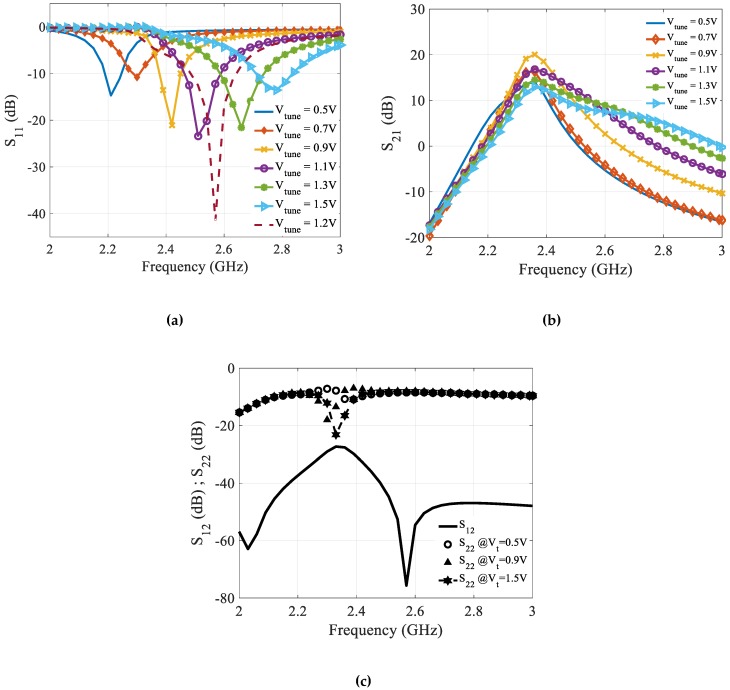
Simulated (**a**) input return loss (S_11_) (**b**) Gain (S_21_) (**c**) Reverse isolation (S_12_) and output return loss (S_22_).

The proposed CTLNA is designed and simulated in MIC process. Keysight ADS and MATLAB are used as simulation tools for CTLNA analysis. The circuit is biased with 1.8 V supply and sinks 9 mA current. As can be seen in Figure 11a, S_11_ achieves a peak minimum for all different values of $V_{tune}$ from 0.5 V to 1.5 V in steps of 0.2 V. It is below −10 dB at each center frequency for the entire tuning range and achieves as low as −40.4 dB at 2.57 GHz at $V_{tune}$ = 1.2 V.

The LNA input matching network has been designed to match to 50 Ω at a particular centre frequency in the tuning range. The calculated 3 dB bandwidth at 2.2 GHz, 2.3 GHz, 2.41 GHz, 2.52 GHz and 2.65 GHz are 20 MHz, 100 MHz, 10 MHz, 10 MHz and 30 MHz, respectively.

Figure 11b shows simulated gain for the designed CTLNA. The LNA gain directly depends on value of loading inductor *L_2_*. However, due to its dependency on α it can be tuned to different frequencies from 2.2 to 2.8 GHz. In addition, the CTLNA gain depends upon $g_{m1}$, $g_{m2}$, $C_{gs1}\cdot C_{gs2}$ source degeneration inductor $L_{s}$, and designed transformer parameters. The LNA achieves a maximum gain of 18 dB at 2.36 GHz in the stipulated tuning range. The minimum gain at 2.2 GHz center frequency is approximately 8 dB. Transistors Q_7_ and Q_8_ in the buffer stage are capable enough to stabilize the LNA and achieve high output impedance.

The output return loss S_22_ is less than -8dB in the tuning range and achieves a peak minimum at center frequency of 2.35 GHz, which is the resonant frequency of output matching network. The reverse isolation S_12_ also remains more than 30dB across the tuning range. Figure 11c shows the variation of S_12_ and S_22_ with frequencies of selected band.

**Figure 12:**
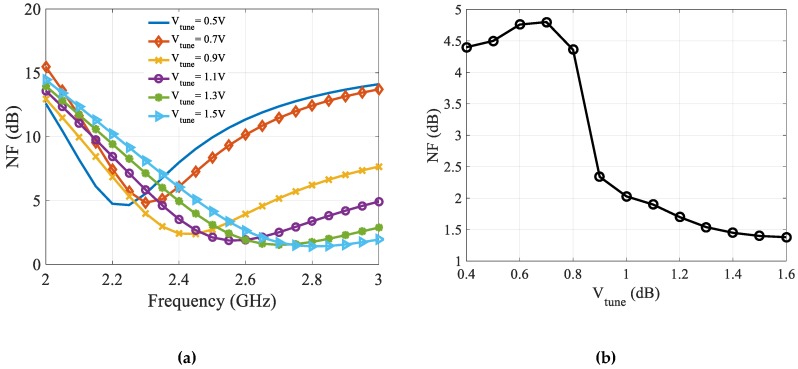
Simulated NF vs. (**a**) Frequency (**b**) *V_tune_*.

Figure 12a,b show simulated NF for designed CTLNA with tuning frequency and *V_tune_*, respectively. It is clear from Figure 12b that minimum NF at each center frequency varies between 1.4 dB to 4.8 dB. NF is a bit higher for 2.2 GHz and 2.3 GHz, which are initial frequencies in the tuning range. However, it is lower than 2 dB at center frequencies ranging from 2.4 GHz to 2.8 GHz.

The LNA stability depends upon the source and the load matching networks, which depends on the frequency of operation. Consequently, the designed CTLNA is supposed to be stable at a particular center frequency while it is unstable at other frequencies. Stability of LNA can be determined by calculating stability factor *K* and stability constant Δ or by plotting stability circles. *K* and Δ are can be mathematically determined using either Rollet’s criteria or Tan’s formulae [24] as:

(35) $$K=\frac{1-{\left|S_{11}\right|}^{2}-{\left|S_{22}\right|}^{2}+{\left|\Delta \right|}^{2}}{2\left|S_{21}S_{12}\right|}$$

(36) $$\Delta =S_{11}S_{22}-S_{12}S_{21}$$

(37) $$K_{t}=\frac{3-2{\left|S_{11}\right|}^{2}-2{\left|S_{22}\right|}^{2}+{\left|\Delta \right|}^{2}-\left|1-{\left|\Delta \right|}^{2}\right|}{4\left|S_{12}S_{21}\right|}$$

**Figure 13:**
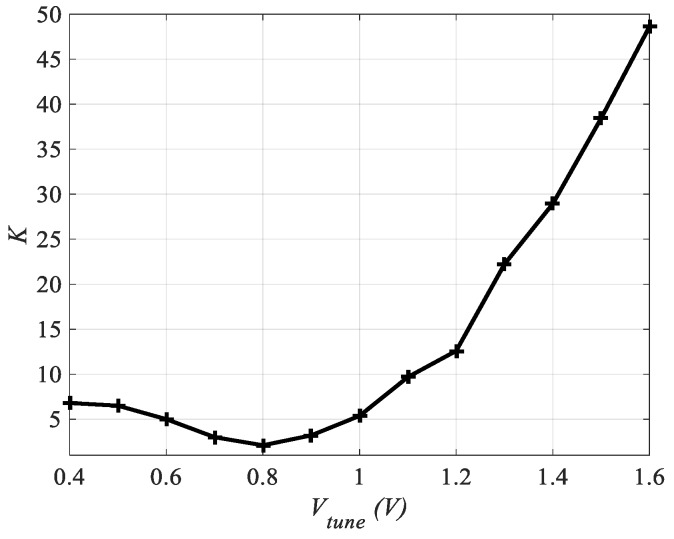
Variation of Stability factor *K* with *V_tune_*.

For the designed LNA, *K* > 1 and |Δ| < 1 at all center frequencies within in the tuning range. *K_t_* > 1 is a single variable criterion to determine the unconditional stability of LNA [24]. Subsequently, the LNA is stable in the entire tuning range. Figure 13 shows variation of *K* with *V_tune_* at different center frequencies.

**Figure 14:**
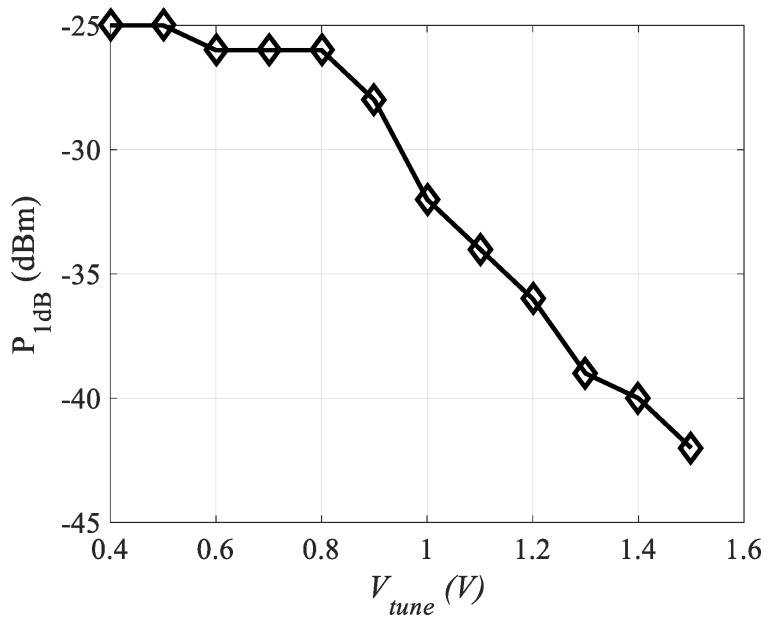
Variation of P_1dB_ with *V_tune_*.

Linearity of LNA is commonly measured by determining 1-dB compression point P_1dB_ and third-order intercept point IP_3_. Non-linearities in the system lead to gain-compression that causes the LNA gain to deviate from the normal curve. P_1dB_ and IIP_3_ calculations have been performed using 1-tone and 2-tone inputs, respectively. A non-linear model of the amplifier is analyzed with a frequency offset of 10 MHz between two tones. The source and load impedances have been set to 50 Ω, while the harmonic frequency was selected to be 2.4 GHz. IP_3_ and P_1dB_ values for the designed CTLNA, range between −15 dBm to −31 dBm and −25 dBm to −42 dBm, respectively. Figure 14 shows the variation of P_1dB_ with *V_tune_* in steps of 0.1V for the proposed CTLNA.

## 6. Conclusions

The design and analysis of an input tuning LNA with transformer based variable inductor matching is presented. The proposed CTLNA can be primarily used for SDR applications, such as green radio networks. The presented design takes advantage of continuous tuning due to magnetic coupling between primary and secondary windings of transformer. This occurs by changing the ratio of currents through primary and secondary windings of the transformer network. To achieve tunability, currents through transformer windings should be in phase. The methodology can be used to further implement a tunable LNA along the frequency band of 2.2 to 2.8 GHz. The design effectively integrates the matching network into an inductively degenerated CS amplifier. The LNA achieves a wideband and tunable gain in the stipulated bandwidth. The input return loss is less than −10 dB and achieves a minimum of −40.4 dB at 2.57 GHz. The NF ranges between 1.4 to 4.8 dB. In addition, mathematical analysis of transformer model, phase shifter and amplification stage are discussed. The proposed technique outlines an idea of continuous tuning that can be implemented to scale the input inductor value for any related application. Table 2 summarizes the simulated performance of designed CTLNA and comparison with previously published works.

## Figures and Tables

**Table 1:** Transformer design parameters.

Parameter	Value
Turns Ratio ‘N’	0.69
Magnetising Inductance ‘*L_tp_*’	2.23 nH
Cross loss resistance ‘*R_c_*’	1000 Ω
Coefficient of Coupling ‘*k*’	0.11
Primary loss resistance ‘*R_t1_*’	0.91 Ω
Secondary loss resistance ‘*R_t2_*’	4.47 Ω
Primary capacitance ‘*C_t1_*’	924 *fF*
Secondary capacitance ‘*C_t2_*’	150 *fF*
Interwinding capacitance ‘*C_t_*’	340 *fF*

**Table 2:** CTLNA performance summary and comparison with previously published works.

Ref.	Freq. (GHz)	S_21_ (dB)	S_11_ (dB)	NF (dB)	IP_3_ (dBm)	V_DD_	Tech.	P_DC_ (mW)
This work	2.2–2.8	7–18	−40–−11	1.4–4.8	−31–15	1.8	MIC	16.2
[16]	1–5	19–27	−18–−5	2.4–3.8	-	1.2	65 nm CMOS	12.1
[5]	1.9–2.4	10–14	−25–12	3.2–3.7	−6.7	1.2	0.13 µm CMOS	17
[12]	2.4–5.4	9.9–22	−14–−30	2.4–4.9	−20.4–−9.7	1	0.13 µm CMOS	3.1–4.6
[13]	0.8–2.5	17–20	−27–−11	3.1–3.6	-	1.8	0.18 µm CMOS	
